# Improving Donor Conversion Rates at a Level One Trauma Center: Impact of Best Practice Guidelines

**DOI:** 10.7759/cureus.891

**Published:** 2016-11-22

**Authors:** Rodrigo F Alban, Bobby L Gibbons, Vanessa L Bershad

**Affiliations:** 1 Surgical Critical Care, Cedars-Sinai Medical Center; 2 Department of Surgical Education, Orlando Regional Medical Center; 3 TransLife Organ Procurement Organization, Orlando Regional Medical Center

**Keywords:** organ donation, organ procurement organization, donor conversion rate, organ donors, transplant surgery, donation champion training

## Abstract

**Background:**

Organ availability is a consistently limiting factor in transplant surgery. A primary driver of this limitation is donor conversion rate, which is defined as the percentage of eligible donors for whom procurement is actually performed. An alternative way to increase organ availability is through improved utilization of organs from donors after cardiac death (DCD). Recently, a concerted, multidisciplinary effort has been made within our system to improve conversion rates and DCD utilization, thus increasing organ availability.

**Study design:**

Retrospective analysis of a prospectively collected database from TransLife, our local organ procurement organization (OPO), as well as the Orlando Regional Medical Center (ORMC) trauma registry, from 2009-2012 (up to 2013 for DCD). During which time, this organization implemented best practice guidelines to improve conversions and DCD utilization. We analyzed yearly conversion rates, DCD donations and population demographics before and after implementation of these policies.

**Results:**

During the study period, donor conversion rates significantly improved from 58% in 2009 to 82% percent in 2012 hospital-wide (P<0.05); and from 50% in 2009 to 81% in 2012 among trauma patients alone (P<0.05). In addition, total organs transplanted increased from 13 to 31 organs (P<0.05) after implementation of best practice guidelines. No significant differences in trauma population demographics were noted during the study period.

**Conclusions:**

Based on our experience, the establishment of best practice policies for referral of potential donors, coupled with programs to educate hospital staff on the existence and importance of these policies, leads to significant improvement in donor conversion rates and increased utilization of DCD donors.

## Introduction

Organ donation and transplantation are critical to improving survival and quality of life in patients with severe organ failure who have failed maximal medical therapy [1]. Organ availability is a consistently limiting factor in transplant surgery. As the number of available donors in a given area remains fairly constant from one year to the next [1], the primary driver of this limitation is the donor conversion rate (DCR), defined as the percentage of potential organ donors (PODs) for whom procurement is performed. Nationally, this rate is approximately 42%. This rate can be artificially improved by changing the way in which PODs are defined [2]. However, this does not actually increase organ availability.

One of the greatest challenges in truly improving DCR, and subsequently increasing the number of organs available for transplantation, is early identification of PODs and involvement of the local OPO [3-4]. Unfortunately, as few as one-third of potential organ donors who have suffered severe traumatic brain injury (TBI) are identified [5].

Equally challenging is the family members’ choice of donation for their loved ones [6]. This is a complex process with many factors at play, including the opinions and attitudes of the hospital care team [7-8], optimal request patterns, and multiple family-engaged discussions with the OPO representative [6,9]. Other variables known to impact the decision-making process include:

- Perception of high-quality care for the POD [10] - Clear understanding of donors after brain death (DBD) and DCD procedures [11] - Temporal separation between discussions of organ donation and notification of the patient’s critical status or death [10] - Requests made in a private setting or made only by highly trained individuals [12-13]

One method that has been shown in recent years to be a viable method for truly increasing the donor pool is the increased use of DCDs [14-15]. To further this, the American Society of Transplant Surgeons released practice recommendation guidelines in 2009 to guide the use of this population [16]. Although organ yield and recipient outcomes are inferior when compared to DBD, it is recommended as a way to expand the donor pool in PODs who do not meet brain death criteria [11,17]. While this successfully increases the number of PODs, this population is still subject to the same challenges that limit donor conversion rates for standard donors after brain death [11,18].

## Materials and methods

We performed a retrospective analysis of prospectively collected data from our local OPO's database (TransLife), as well as the ORMC trauma registry from 2009-2012 (up to 2013 for DCD). The OPO database contains data regarding referral and outcomes of all donors, and the trauma registry prospectively collected demographic and injury severity data for all patients admitted to the trauma service during that time.

Data collected included total number of PODs, defined as any patient admitted to the intensive care unit (ICU) requiring mechanical ventilation and with risk of imminent death, DCRs (fraction of potential donors who become actual donors), DBDs, DCDs, and total numbers of organs donated before, during, and after the intervention period. Additionally, trauma patients were analyzed separately as a significant portion of PODs were trauma patients identified within our multidisciplinary ICUs. We analyzed trauma population demographics, including injury severity score (ISS), abbreviated injury score (AIS) head, Glasgow Coma Scale (GCS), and age and length of stay, for both DBD and DCD donors, to determine if increases in the DCR were correlated to improved practices.

Statistical analysis was done using SPSS Statistics, Version 17.0 (SPSS, Inc., Chicago, IL). Data are reported as mean. Categorical variables were analyzed with a Fisher's exact test and continuous variables with a Mann-Whitney U test. A P-value of less than 0.05 was considered significant. IRB exemption was obtained.

## Results

The task force developed to oversight the donation process was started in 2007 and over the next several years, several policy changes were implemented to improve DCR. The evaluation phase for our data was available from 2009, and the most significant changes, including implementation of donation champion training, were instituted in 2011, including dedicated training specific to ICU and emergency department (ED) nursing staff (Table 1).

**Table 1:** Policy Changes to Improve Donor Conversion Rates

Year	Policy Changes to Improve Donor Conversion Rates				
2007	First ORMC donation collaborative team (Team ORLANDO) is held, made up of physicians, nurse leadership, chaplaincy, nurse educators, bedside nurses, and TransLife staff.				
2008	Task force formed within Team ORLANDO to look at approach and consents in order to understand low conversion rate.		Residents recognized as inappropriately approaching families. Issue discussed within Team ORLANDO and problems felt to include lack of education for all residents.		
2009	Critical check approved for the brain-injured patient. Provides guidance for staff to maintain patients for brain death testing and potential donation through proper management.		Donation education series proposed to Team ORLANDO to allow team members dedicated time to learn about the donation process.	Decided that 2010 hospital team members, not TransLife, will chair Team ORLANDO.	Attendings and residents received education regarding donation request.
2010	First Donation Champion Training series held with two sessions; ICU RNs only.	Began use of End of Life Specialist term for TransLife representatives to address “What’s next?” question from families.		Second Donation Champion Training series is offered with two sessions.	Assigned RNs who have been through Donation Champion Training to POD patients.
2011	Donation Champion Training session offered. Beginning in 2011, sessions offered twice a year in spring and fall. Full ICU staff and ED staff now included.				
2012	ED nursing champion named.		Availability of TransLife lab vials in ED and posted signs in ICUs to facilitate immediate lab draws and promote awareness.		

Over the study period, there were a total of 187 PODs in the DBD population. Of these, 124 (66.3%) were admitted to the trauma service and entered into our database. The average age for this trauma cohort within the DBD group ranged from 31 to 37 years old, and their injury severity scores were high, ranging from 18.7-24.5 (Table 2).

**Table 2:** Donors After Brain Death - Trauma Patient Cohort ISS= Injury severity score AIS= Abbreviated injury score GCS= Glasgow Coma Scale HLOS= Hospital length of stay ICU LOS= Intensive care unit length of stay

Year	ISS	AIS Head	GCS	HLOS	ICU LOS	Age
2009	23.2	3.6	3.7	3	3.5	37.4
2010	19.6	3.8	3.5	2.7	3.2	35
2011	18.7	3.4	3.5	2	3.3	32
2012	24.5	4	3.5	2.3	3.4	31

**Figure 1:**
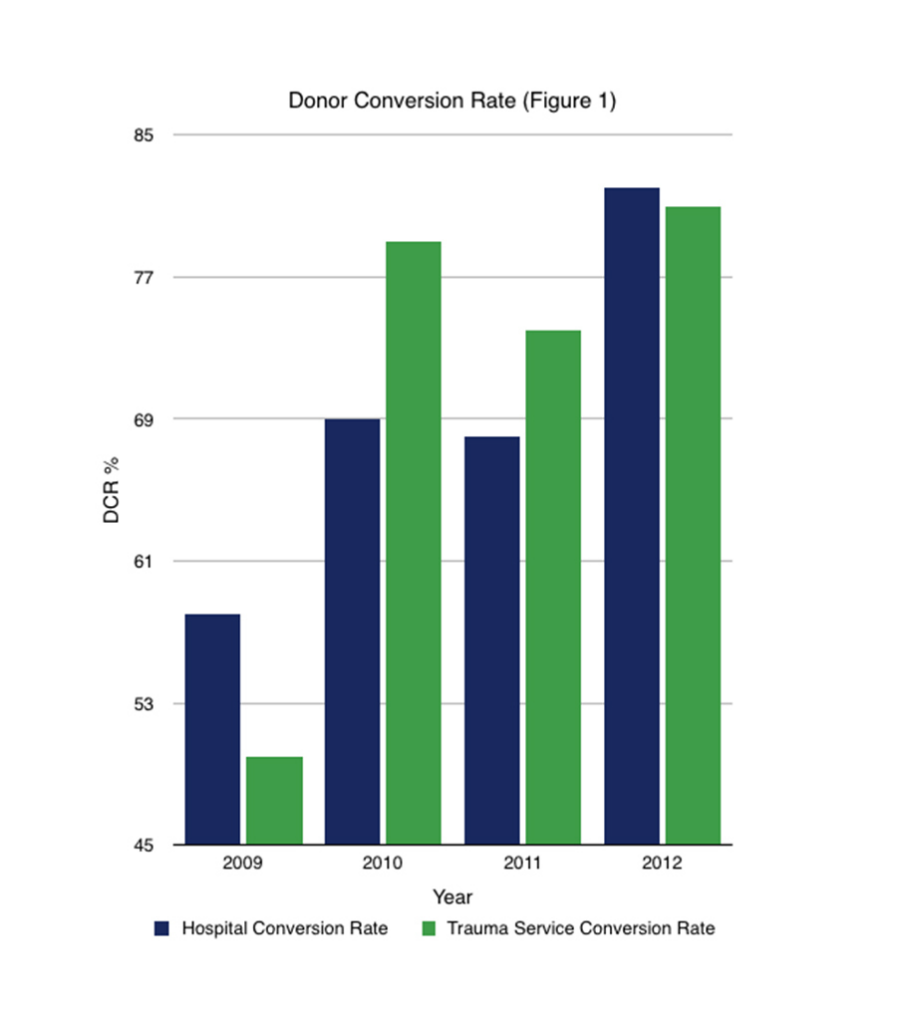
Donor Conversion Rate

Between 2009 and 2012, DCR for DBDs improved significantly: from 58% in 2009 to 82% percent in 2012, hospital-wide (P<0.05); and from 50% in 2009 to 81% in 2012 among trauma patients alone (P<0.05) (Figure 1).

This resulted in a total of 89 donors for the trauma service with an average of 4.02 organs per donor (Table 3). 

**Table 3:** Organs per Donor

Year	Kidney	Liver	Heart	Lung	Pancreas	Intestine	Total Used
2009	2	0.8	0.3	0.5	0.4	0	4
2010	1.68	0.84	0.4	0.32	0.16	0.04	3.44
2011	1.68	0.84	0.4	0.32	0.16	0.04	3.44
2012	1.86	0.86	0.43	0.66	0.29	0	4.09

**Figure 2:**
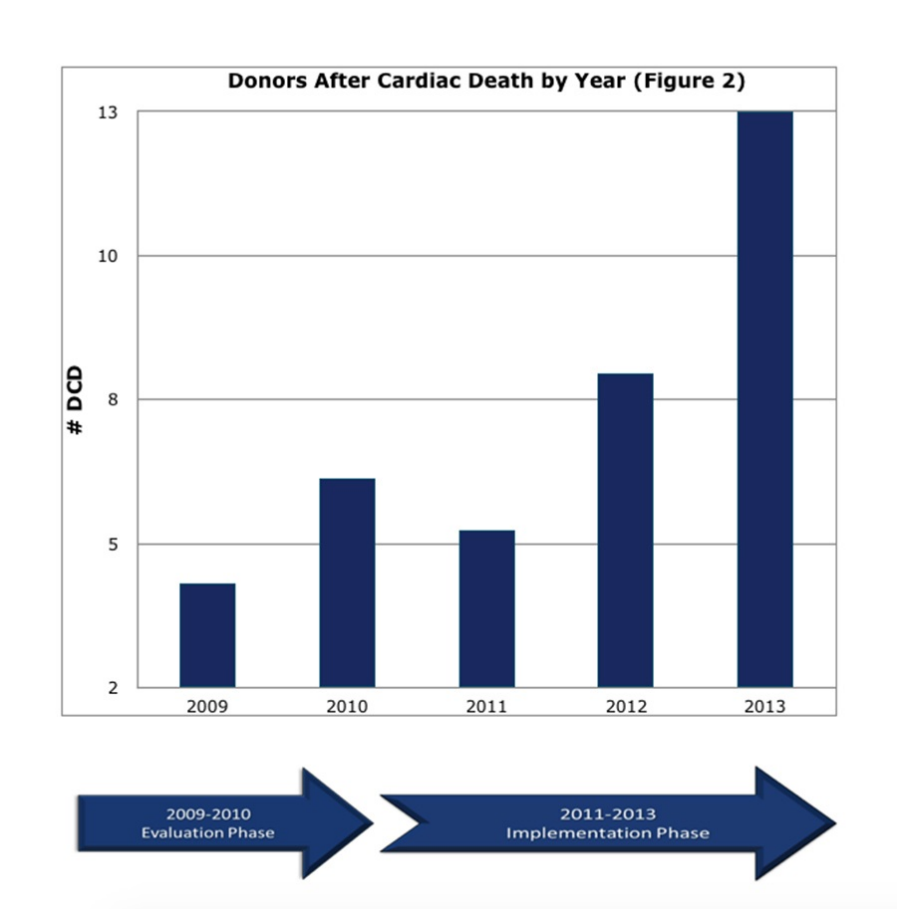
Donors After Cardiac Death by Year

Over the same study period, a total of 10 DCDs were identified before implementation: four in 2009 and six in 2010. This is compared to a total of 26 after the institution of these collaborative practice processes: five in 2011, eight in 2012, and 13 in 2013 (Figure 2).

This represents a 2.6-fold increase in the number of DCD procurements performed at our institution. Concurrently, increases in DCD rates resulted in an increase in organs procured from 13 in 2009 to 31 in 2013 (P<0.05) (Table 4).

**Table 4:** Donors After Cardiac Death - Trauma Patient Cohort ISS= Injury severity score AIS= Abbreviated injury score GCS= Glasgow Coma Scale ICU LOS= Intensive care unit length of stay

Year	ISS	AIS Head	GCS	Total Organs Donated	ICU LOS	Age
2009	28.3	3.3	4.3	13	3.3	37
2010	21.5	4.2	3	16	3.3	35
2011	20.6	3.6	3	12	5.4	32
2012	21.4	3.4	4.3	19	6.3	31

Prior to ED staff participating in Donation Champion Training, one eligible donor was referred from the ED between 2009-2011. In 2012, after training was expanded to include ED staff, twelve PODs were referred directly from the ED (P<0.05).

## Discussion

Over the course of the study period, we showed clear improvements in both the hospital-wide and trauma service-specific conversion rates. In addition, we saw a clear increase in the number of DCD donations. Within the trauma population, the only significant change in demographics noted was a decrease in mean age among PODs. While younger age has been correlated with increased donation rates [9], we did not observe this trend. In fact, in every year except 2012, the mean donor age was higher than the mean age for those who declined donation.

While we can clearly show improvements in DCR and increases in DCD procurements over a period that correlates with changes made to our practices, a significant limitation of our study is the inability to quantify the relationship between the two. Based on the timing, the known relationship between provider attitudes [7-8], and timeliness of referral [9], we believe that the most significant change is our Donation Champion Training series (Table 1). This series reached the greatest number of individuals, and the largest improvement was noted after the first large series. Staff members who completed the training series noted a positive change in their attitude toward the organ donation process. We were also able to document a significant change in the referral pattern from our emergency department staff once they were included in this training.

Health care professionals’ attitudes toward the organ donation process prove to be a major component in organ procurement [7]. Increasing the consent rate is the determining factor in increasing the rate of organ donation in the demographic of brain-dead potential donors [1]. As a means of increasing consent, some states have implemented electronic means for the living to document their organ donation requests, and other institutions have considered financial gifts [1]. However, our Donation Champion Training proved to be most effective in gaining consent to improve DCR. Changing health care professionals’ attitudes toward the organ donation process has been a key component of our success. More positive attitudes toward organ donation correlated with an increase in the number of staff members requesting donation because they believe donation will help the family. As our findings show, this positive belief surrounding the organ donation process resulted in increased consent to donation and an improved DCR.

We feel that Team ORLANDO was equally instrumental in the improvement process. The routine analysis of our POD cases and continuous brainstorming of methods for improvement set us on the path of sequential improvement that culminated with the Donation Champion Training and significant improvement in our DCR.

## Conclusions

Based on our experience, there is no single path to improving donor conversion rates. Stepwise improvements, including the establishment of best practice guidelines for referral of potential donors, coupled with programs to educate hospital staff on the existence and importance of these policies, leads to more timely referrals and a significant improvement in DCR [19]. Team ORLANDO continues to meet monthly to review all potential donor cases and evaluate our practices as we strive for continued improvement in our donation process.
